# Death in the EU/EEA from autochthonous human rabies, Romania, July 2025: a call for action

**DOI:** 10.2807/1560-7917.ES.2025.30.43.2500794

**Published:** 2025-10-30

**Authors:** Mihnea Hurmuzache, Maria A. Gradinaru, Florica Bărbuceanu, Răzvan Moțiu, Rodica Popescu, Andrada Lutic, Thomas Müller, Conrad M. Freuling, Vlad Vuta

**Affiliations:** 1 Clinical Hospital of Infectious Diseases, Iasi, Romania; 2 Institute for Diagnosis and Animal Health, Bucharest, Romania; 3 Faculty of Veterinary Medicine, Bucharest, Romania; 4 National Institute of Public Health, Bucharest, Romania; 5 Iasi County Public Health Directorate, Iasi, Romania; 6 Friedrich-Loeffler-Institute, Greifswald - Insel Riems, Germany

**Keywords:** human rabies, encephalitis, zoonoses, Romania, one health, post-exposure prophylaxis, animal bite

## Abstract

We report a confirmed autochthonous human case of classical rabies in Romania involving an individual in their mid-40s from Iași county, who was bitten by a free-roaming dog in February 2025. The case did not receive post-exposure prophylaxis (PEP) and died from rabies in July 2025. This event highlights critical gaps in rabies prevention, the importance of timely PEP, and the need for continued vigilance in rabies surveillance and public health communication.

Rabies is a zoonotic viral disease with 100% case fatality once clinical symptoms appear [1]. Globally, terrestrial rabies caused by rabies virus (RABV) has been mostly eliminated in domestic dog populations of high-income countries, but sporadic cases still occur due to insufficient prophylaxis following exposure from wildlife reservoirs or spillover hosts [1]. Here we describe a confirmed autochthonous human rabies case in Romania in 2025, which acquired RABV through an infected dog.

## Case presentation

In February 2025, a free-roaming dog entered the property of a resident in their mid-40s of Iași county, Romania, and bit this individual on the hand. The bite was managed with local wound care and a short course of antibiotics. Rabies post-exposure prophylaxis (PEP) was suggested by the local physician but declined by the patient, who around mid-June developed symptoms of confusion and agitation. Due to a progressively worsening neurological condition, the patient was admitted to a psychiatric hospital 4 days later. The course remained unfavourable, with fever (body temperature ≥ 38.5˚C) and hypersalivation developing, and the patient lapsing into coma 5 days after admission.

A neurological computed tomography (CT) scan showed no significant abnormalities. Lumbar puncture revealed a cerebrospinal fluid cell count of 19 cells/mm^3^ (normal range: 0−5 cells/mm^3^). Encephalitis was suspected, and the patient was transferred to the intensive care unit (ICU) of the Hospital of Infectious Diseases in Iași. After further clinical evaluation, rabies was only suspected when the family reported the prior dog bite. The patient remained in the ICU of the infectious disease clinic until succumbing to the infection 34 days post symptom onset after more than 3 weeks of intensive supportive care during hospitalisation.

At the Institute for Diagnosis and Animal Health Bucharest, the clinical diagnosis, which was consistent with classical rabies encephalitis, was confirmed by intra-vitam laboratory tests, i.e. reverse-transcription (RT)-PCR of cerebrospinal fluid (CFS) and saliva samples. In the same laboratory, postmortem analysis of brain material by direct fluorescence antibody tests (dFAT) and RT-PCR detected RABV. Laboratory results were also confirmed at the World Health Organization (WHO) Collaborating Centre for Rabies Surveillance and Research at the Friedrich-Loeffler-Institut (FLI), Riems, Germany. 

## Epidemiological context

In Europe, dog-mediated rabies was eliminated during the mid-20th century and in most European Union (EU) countries fox-mediated rabies has been successfully controlled through extensive oral rabies vaccination (ORV) campaigns [2,3]. Between 1977 and 2025, 285 human rabies cases were reported in Europe, primarily from the Russian Federation (n = 189), Ukraine (n = 13), and Georgia (n = 12). In the last decade (2014−2025), a total of 15 human cases were detected in the EU/European Economic Area (EEA). Of these, 13 were travel associated, and one was caused by a bat lyssavirus other than RABV. For the remaining one, the diagnostic results remain questionable (Table).

**Table:** Human rabies cases detected in the European Union/European Economic Area, 2014−2025 (n = 15)

Year	EU/EEA total reported cases	Reporting country (diagnosis/notification)	Status or exposure location when known	Reference
2025	3	Spain	Exposure in Ethiopia	[12-14]
		United Kingdom	Exposure in Morocco	
		France	Under investigation	
2023	1	France	Exposure in Morocco	[15]
2022	1^a^	Romania	Locally acquired (Romania)	[11]
2019	5	Italy	Exposure in Tanzania	[16]
		Latvia	Exposure in India	
		Spain	Exposure in Morocco	
		Norway	Exposure in Philippines	
		France	Locally acquired EBLV-1 (bat) (France)	
2018	1	United Kingdom	Morocco	[17]
2017	1	France	Exposure in Sri Lanka	[18]
2014	3	Spain	Exposure in Morocco	[19]
		France	Exposure in Mali	
		the Netherlands	Exposure in India	

EBLV-1: European bat lyssavirus 1; EU/EEA: European Union/European Economic Area.

^a^ Case presentation and diagnostics are questionable.

In Romania, which borders rabies-endemic countries to the east (Moldova) and to the north (Ukraine), ORV campaigns should control rabies within the country and are also designed to establish a vaccination barrier (‘*cordon sanitaire’*) to prevent the reintroduction of the disease into rabies-free areas of the EU [3,4]. The standard ORV strategy involves biannual bait distribution in autumn and spring. However, the interruption of ORV campaigns targeting foxes after 2020 — with a total of seven baiting campaigns missed — has led, since 2021, to a resurgence of rabies cases in wild and domestic animals [5-7]. From January to September 2025, 48 rabies cases were identified in such animals across seven counties, of which 30 were in Iași (Figure). This persistence in the animal reservoir poses a direct human risk, especially in rural areas with limited access to veterinary and medical care.

**Figure:**
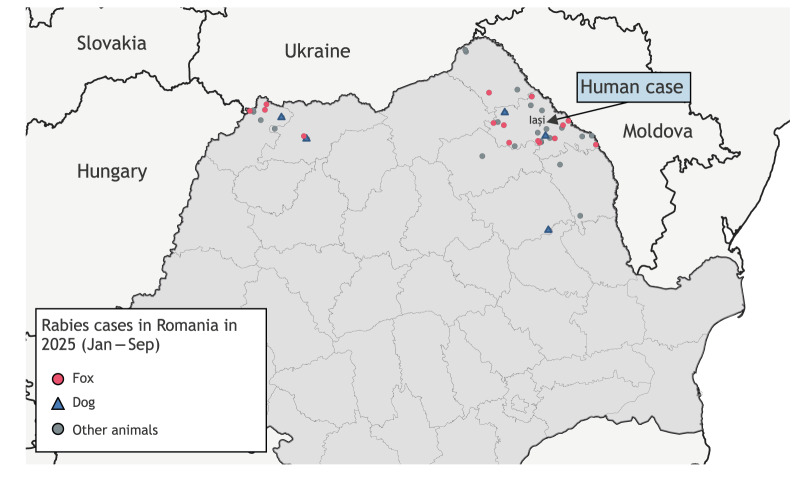
Geographical locations of rabies cases in Romania^a^, January–September 2025 (n = 48)

^a^ Geographical location of rabies cases in Romania as reported to the Rabies Bulletin Europe [6].

## Control measures

After confirmation of the human rabies case in Iași, epidemiological investigations reviewed all dog bite presentations recorded by physicians across the county, with no additional suspected cases occurring near or around the same period (late February 2025). The Iași Public Health Directorate notified and officially informed all family physicians in the county, as well as local authorities including mayors, the police, hospitals, the ambulance service, and the prefecture, about the risk of rabies virus infection among the population and the urgent need to vaccinate individuals who had been in contact with animals suspected of having rabies. 

Veterinary authorities in Iași county implemented several control measures according to national legislation. Stray dogs were captured and taken to animal shelters, while epidemiological investigations identified unvaccinated or recently vaccinated cats and dogs that had been exposed to infected animals; these were subsequently euthanised to prevent further transmission. Potentially exposed susceptible animals were isolated for 30 days, and any of these showing clinical signs compatible with rabies were euthanised. Mandatory vaccination campaigns for cats and dogs were carried out, passive surveillance which involves post-mortem testing of symptomatic or deceased wild and domestic animals using FAT and RT-PCR was strengthened, and hunts targeting foxes and jackals were organised to reduce potential reservoirs.

## Discussion

The fatal autochthonous human case in 2025 illustrates that rabies, though largely eliminated from the EU/EEA, remains a persistent threat when control programmes, e.g. ORV campaigns, and vigilance wanes.

Rabies is fully preventable through timely administration of PEP after exposure [8,9], yet the patient described here declined receiving vaccination or immunoglobulin following a dog bite that was clearly high-risk, given the epidemiological evidence of ongoing rabies circulation in the north and northeast of Romania (Figure). That the patient did not recognise the need for prophylaxis at the time of injury may reflect that people in endemic areas are not fully conscious of the risk of rabies. In turn, this possibly signals a critical public health failure in communicating the risk. The late recognition of rabies by healthcare professionals, once symptoms appeared, with the patient first admitted to a psychiatric facility, further underlines how easily rabies may be overlooked in settings where dog-mediated transmission is assumed to be eliminated. Thus, strengthening awareness among frontline healthcare workers is as important as educating the general public.

In recent years, animal rabies cases in the EU typically have resulted from cross-border transmissions from neighbouring rabies endemic countries [6]. In Romania, increasing numbers of rabies cases in both wild and domestic animals have been reported since ORV of foxes was interrupted in 2021 [6,7]. An audit by the EU identified procurement or administrative issues that hampered the implementation of this programme [10]. The resurgence of RABV in the animal reservoir not only reintroduces risks to human and animal populations but also threatens progress towards regional elimination. Similar challenges are faced by other EU-countries along the eastern border of the EU [5]. 

Additionally, human rabies in Romania [11] has not been systematically reported to the WHO Rabies Bulletin Europe or to international organisations such as the European Centre for Disease Prevention and Control (ECDC) or the European Food Safety Authority (EFSA). The failure to notify cases to these international bodies hinders coordinated responses and undermines the shared European objective of rabies elimination.

Addressing these gaps requires an integrated response. Public awareness campaigns should stress that every animal bite constitutes a potential rabies exposure and requires immediate medical evaluation. Health authorities must guarantee access to PEP, including vaccines and rabies immunoglobulin, across the country, particularly in endemic areas (Figure). Equally urgent is the reinstatement of sustained rabies control measures in animals; the reintroduction of ORV programmes targeting foxes, together with strengthened vaccination coverage among domestic dogs, is essential to break the cycle of transmission. Management of free-roaming and stray dog populations must be incorporated into these initiatives to reduce the interface between humans, domestic animals, and wildlife. Ultimately, the human case in 2025 demonstrates that rabies control cannot be treated solely as a veterinary issue or as a clinical problem for human medicine. Instead, it demands a One Health approach that bridges human, animal, and environmental health sectors. At least for this case the ‘One Health’ or ‘One Medicine’ approach was used by networking between human medicine, veterinary medicine and research institutions. The tragedy in Iași county is a reminder that rabies, though 100% fatal, is also 100% preventable through vaccination or PEP. Allowing preventable human deaths to occur in the EU/EEA due to a disease long considered under control is unacceptable.

A limitation of the current study is that the dog implicated in this fatal case was never identified or tested, precluding definite confirmation of the source of infection. Consequently, the possibility of additional, unrecognised exposures among humans and animals cannot be excluded, and administration of post-exposure prophylaxis (PEP) was substantially delayed. Nevertheless, no further human cases have been reported to date.

## Conclusion

The case presented here represents a serious event in Europe’s rabies landscape, underscoring persistent vulnerabilities. It should serve as an alert for Europe and for Romania, highlighting the urgent need to restore and sustain rabies prevention programmes, reinforce public and professional awareness, and maintain transparent international reporting. Only through continuous vigilance and coordinated action can Europe prevent rabies from regaining a foothold and ensure that no further lives are lost to this ancient but entirely avoidable disease.

## Data Availability

Epidemiological and clinical data are available upon reasonable request from the corresponding author.
